# Secondary prophylaxis in rheumatic fever: is it time to change?

**DOI:** 10.1186/1824-7288-41-S2-A71

**Published:** 2015-09-30

**Authors:** Andrea Taddio, A Pirrone, Serena Pastore, Loredana Lepore, Caterina Di Battista, Gabriele Simonini, Luciana Breda, Rolando Cimaz

**Affiliations:** 1 Institute for Maternal and Child Health - IRCCS “Burlo Garofolo” – Trieste, Italy; 2 University of Trieste, Trieste, Italy; 3 Department of Paediatrics, University of Chieti, Chieti, Italy; 4 AOU Meyer, Florence, Italy

## Background

Carditis and rheumatic chronic heart disease are the most serious complications of Acute Rheumatic Fever. Nowadays prevention of recurrent episodes of group A β-hemolytic streptococcal pharyngitis is the most effective method to prevent the development of severe rheumatic heart disease. However the evidence of these guidelines are weak and result from studies conducted more than 50 years ago [1]. To detect rate of Carditis related to Acute Rheumatic Fever found at follow up and to find a relationship with clinical data at diagnosis and compliance to prophylaxis.

## Material and methods

This is a multicentre retrospective study conducted among 117 pediatric patients admitted with diagnosis of rheumatic carditis. We analysed the presence of carditis at diagnosis and at follow up comparing it with the number of infection recurrences and with the level of compliance of the patients to antibiotic prophylaxis. Compliance to antibiotic prophylaxis was evaluated individually with a questionnaire. The association between recurrences and compliance to therapy and between recurrences of streptococcal infections and carditis at follow up was also analyzed. Data were analysed using Fisher exact test.

## Results

We examined 117 pediatric patients with rheumatic fever carditis. The median age of the patients at diagnosis was 9 years (6-11 years). The median age at follow up was 15 years, and the data at follow up were taken at a median time of 6,8 years from diagnosis. The data show that carditis at follow up was associated with the presence of carditis at diagnosis (p<0.000) and not with the level of compliance to antibiotic prophylaxis (p=NS). Also there was no statistically significant association between recurrences of infections and good level of compliance to therapy (p=NS) and between number of recurrences and pesence of carditis at follow up (p=NS).

## Conclusions

We observed that the risk to develop carditis at follow up in pediatric patients with Acute Rheumatic Fever is independent from the compliance to antibiotic prophylaxis and the number of infection episodes caused by group A β-hemolytic Streptococcus while it seems related to the presence of carditis at time of diagnosis.
